# Congenital Muscular Dystrophy Due to Merosin Deficiency: Report of a New Mutation

**DOI:** 10.7759/cureus.39988

**Published:** 2023-06-05

**Authors:** Wilmer Santiago Herrera Malpica, Fernando Ortiz-Corredor, Diana Sanchez Peñarete, Paula Vanessa Muñetones Hernández

**Affiliations:** 1 Physical Medicine and Rehabilitation, Universidad Nacional De Colombia, Bogotá, COL; 2 Genetics, Instituto Roosevelt, Bogotá, COL; 3 General Medicine, Universidad Nacional De Colombia, Bogotá, COL

**Keywords:** phenotype, ultrasonography, lama 2 gene sequencing, hypotonia, merosin, congenital muscular dystrophy

## Abstract

Congenital muscular dystrophy due to merosin deficiency is one of the most common congenital muscular dystrophies. It is characterized by a LAMA2 gene mutation and causes varied clinical symptoms depending on the type of presentation. In this case report, we identified the importance of the medical history and the autosomal recessive expression, which compromises the sequencing of the LAMA2 gene, with a mutation variant c. 1854_1861dup (p. Leu621Hisfs*7), in homozygosity not described so far. As well as the phenotypic characteristics of the evidenced mutation.

A 13-year-old patient presented with a clinical history that began at 18 months of age. According to the mother, the patient had a delay in neurological development and could not walk since he was 7. In addition, contractures were observed in the lower extremity, elbows, and fingers of both hands. The patient also had scoliosis, bilateral hip dysplasia, and sleep apnea-hypopnea syndrome. However, cognitive function was unaffected. Extension studies revealed elevated creatine kinase levels, electromyography indicated muscle fiber involvement, and brain resonance imaging showed a hyperintense lesion at the periventricular level along with symmetrical supratentorial findings. Immunohistochemical studies of merosin showed incomplete reactivity and gene sequencing revealed evidence of a LAMA2 mutation: c. 1854_1861dup (p. Leu621Hisfs*7), in homozygosity.

Congenital muscular dystrophy caused by merosin deficiency is characterized by the absence of laminin alpha-2. The clinical manifestation of this disease is a severe phenotype, mainly due to the early onset of the disease. In patients with mutations in the LAMA2 gene, the absence or partial reduction of laminin alpha-2 staining may allow some degree of ambulation, as it could indicate a partially functional protein. To complement clinical, immunohistochemical, and pathologic findings, ultrasound can be used as a potential tool for monitoring or assisting in the diagnosis of individuals with congenital muscular dystrophy. In this study, we performed sequencing of the LAMA2 gene, which revealed a homozygous c. 1854_1861dup (p. Leu621Hisfs*7) mutation. In addition, we describe the phenotypic features associated with this specific mutation.

## Introduction

Congenital muscular dystrophy (CMD) resulting from merosin or laminin alpha-2 deficiency is a prevalent cause of CMD [1]. Diagnosis of this condition typically involves assessing the clinical presentation, measuring elevated levels of creatine kinase, conducting brain magnetic resonance imaging, and performing biopsy examinations, which include conventional histology and immunohistochemistry. Ultrasonography has emerged as a valuable tool in assessing muscle involvement and changes in muscle tissue echogenicity among patients with congenital muscular dystrophies.

CMD is a group of muscular dystrophies characterized by an early onset [2]. The clinical presentation of this disorder can exhibit significant variation, depending on the age of onset, with early-onset cases generally demonstrating more severe symptoms compared to those with a later onset. Nevertheless, predicting the precise clinical phenotype based on the expressed levels of laminin alpha-2 protein remains uncertain [3].

In this case report, we present a novel mutational variant, designated as c. 1854_1861dup (p. Leu621Hisfs*7) in homozygosity, accompanied by specific clinical characteristics. These characteristics include predominantly proximal weakness with preserved distal strength, upper and lower limb contractures leading to deformities, neuromuscular scoliosis, and the absence of swallowing or communication disorders. Cognitive impairment was also not observed in the patient. We utilized ultrasonography to visualize the involvement of muscle tissue and track its progression during follow-up visits.

## Case presentation

The patient in question is a 13-year-old who presented clinical symptoms starting at 18 months of age. These symptoms initially manifested as a neurodevelopmental delay. The patient achieved head support at six months, and attained a sitting position at eight months, but did not crawl. At two years of age, contracture was observed in the left lower limb, which progressively extended to the right lower limb, elbows, and fingers of both hands. The patient was never able to walk, and progressive scoliosis has also been noted. As evidence, a motor function measures 20 (MFM20) was performed at five years of age. The total score was 55%. Domain 1 was the most compromised (7.6%). However, in domains 2 and 3 the scores were 94% and 76%, respectively. This indicated a severe disability for basic transitions, bipedal, and gait, but he was able to hold himself in a chair without any support.

The patient's medical history includes bilateral hip dysplasia, sleep apnea-hypopnea syndrome, and vitamin D deficiency. There is a family history of a 17-month-old sister with neurodevelopmental delay, and a genetic study revealed a LAMA2 alteration (duplication, homozygous). The sister did not exhibit any fine motor skill alterations, had normal oral language development, and showed no issues with swallowing. During the physical examination, the patient was found to be wheelchair-bound and propelled by the mother. There was a right convexity thoracolumbar scoliotic curve, and the patient's upper limbs exhibited retractions that hindered the full extension of the elbows. The patient can raise her arms above her head with compensations.

Muscle strength assessment revealed 3+/5 bilateral strength in the proximal deltoid muscles, 4-/5 bilateral strength in the biceps, 4/5 bilateral strength in the extensor carpi radialis, and 4+/5 bilateral strength in the tibialis anterior and gastrocnemius muscles. The lower limbs displayed 35º flexion deformity in the hips and 60º flexion deformity in the knees. Additionally, the iliopsoas muscle exhibited 3+/5 bilateral strength, while the quadriceps demonstrated 4/5 bilateral strength. In self-care, the child eats without help, handles a spoon and fork, can brush her teeth, has difficulty combing her hair, and can wash her face. She can put on and take off a shirt, handles buttons, and zippers. Cannot take off or put on pants and needs assistance on the toilet. Although able to sit up, needs assistance with all transfers.

**Figure 1:**
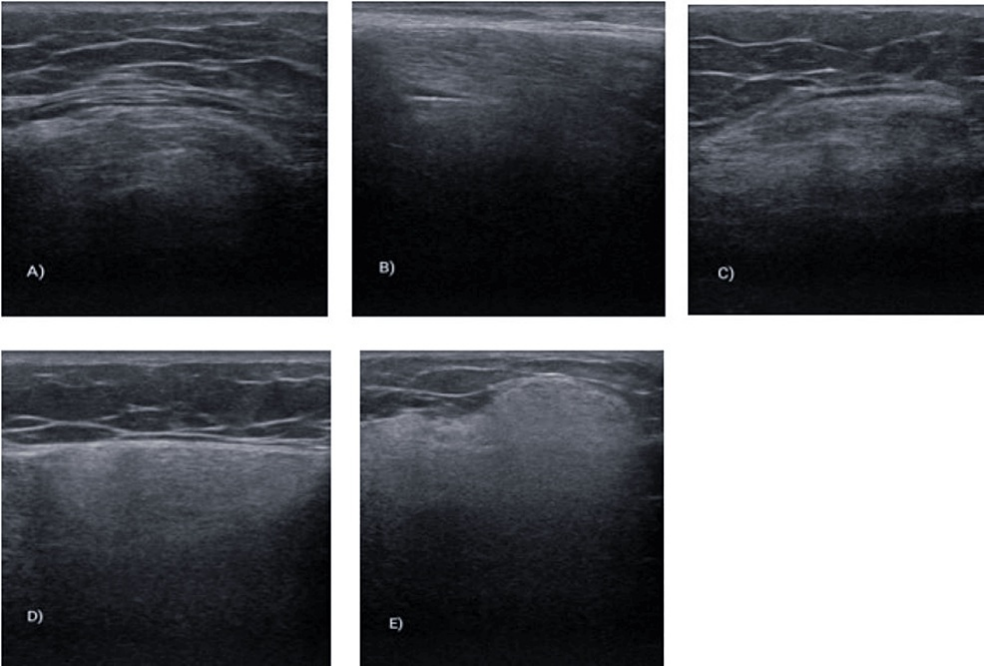
Ultrasonography Esaote equipment with 18 MHz SL2325 linear array transducer shows (A) rectus femoris, (B) Psoas, (C) medial gastrocnemius, (D) anterior tibialis, and (E) biceps brachialis.

Laboratory testing, imaging studies such as ultrasonography (Figures 1A-1E), neurophysiological examinations, biopsies, and genetic studies were performed. The findings revealed elevated creatine kinase levels, a hyperintense lesion in symmetric supratentorial periventricular white matter on brain MRI, a normal echocardiogram, abnormal electromyography (EMG) consistent with primary muscle fiber disease, and muscle biopsy indicating a dystrophic pattern with marked changes of chronicity (Table 1); abnormal merosin testing showed incomplete reactivity. Additional genetic studies confirmed LAMA2 gene sequencing: c. 1854_1861dup (p. Leu621Hisfs*7) in homozygosity.

**Table 1:** Results of labs and images

Labs and images	Results
Creatine kinase	1,660 UI/L
Brain MRI	Hyperintense lesion in symmetric supratentorial periventricular white matter
Muscle biopsy	Dystrophic pattern with marked changes of chronicity
Immunohistochemistry	Does not show complete and intense reactivity for merosin
Transthoracic echocardiogram	Normal, FEVI 68%
Spine X-ray	Right convex scoliosis of the lower thoracic spine with a coob angle of 49° and a left convex scoliosis of the lumbar spine with a coob angle of 19°.
Pelvis X-ray	Dysplastic configuration of the acetabulum with increased angle of acetabular inclination.
EMG	Polyphasic potentials of low amplitude and short duration
Panel for Limb-Girdle Dystrophy	Normal
LAMB2 gene sequencing	Normal
LAMA2 gene sequencing + analysis of duplication deletions (CNV) of the LAMA2 gene	c. 1854_1861dup (p. Leu621Hisfs*7), homozygosity

Details of the variant are the sequence change that inserts eight nucleotides into exon 13 of the LAMA2 mRNA (c.1854_1861dupACGTGTTC), causing a frameshift at codon 621. This creates a premature translation stop signal (p. Leu621Hisfs*7), which is expected to result in the absence or interruption of the protein product.

## Discussion

Skeletal muscle relies on muscle fibers as its functional units, with the junction between the sarcolemma and basement membrane playing a crucial role in muscle fiber stability and signal transduction [1]. The basement membrane, along with extracellular matrix proteins, surrounds the muscle fibers and provides resistance against the mechanical force generated during contraction. Mutations in basement membrane proteins can lead to muscular dystrophies of varying severity and onset time [2]. In contrast to myopathies, which result from functional defects in the contractile apparatus, dystrophies primarily involve abnormalities in the muscle membrane [2].

Type 1A CMD is characterized by merosin deficiency, an autosomal recessive disease caused by the absence of laminin alpha-2, a protein involved in the connection between the cytoskeleton and extracellular matrix [3]. This condition arises from a mutation in the LAMA2 gene, located on chromosome 6q22-q23 and comprising 65 exons [4].

The clinical spectrum of this mutation encompasses a wide range of symptoms, from patients who can walk to those who are unable to sit or stand. The severity of the disease is more pronounced in individuals with early-onset presentations, whereas late-onset cases exhibit milder symptoms. Overall, CMD due to merosin deficiency accounts for approximately 30% of all patients with CMD [5].

In early-onset clinical presentations, patients experience hypotonia, muscle weakness, inability to walk independently, proximal joint contractures, respiratory failure with recurrent infections, and normal intellectual development. On the other hand, late-onset clinical presentations involve proximal muscle weakness, delayed motor milestones, and an inability to ambulate [5].

Most patients manifest the disease within the first year, accompanied by elevated creatine kinase levels ranging from 940 to 4000 U/l [6]. Magnetic resonance imaging reveals hyperintense areas in the periventricular and subcortical white matter [7]. A case series report demonstrated substantial variation in dystrophic muscle biopsies and clinical phenotypes among seven patients, highlighting the presence of laminin alpha-2 deficiency, which can be identified through dystrophic changes in muscle biopsy with routine staining for dystrophin [3].

Previous studies have identified additional manifestations such as hip dislocations, external ophthalmoplegia, and cardiomyopathies. Weight gain, facial weakness, scoliosis, and multiple contractures are among the most prominent features. Focal motor or visual seizures may occasionally occur, along with the presence of polymicrogyria. Genetic analysis using multiplex ligation-dependent probe amplification (MLPA) has identified two mutations in the LAMA2 gene in the majority of patients, although some individuals exhibit only one detected mutation. Both homozygous and heterozygous mutations have been observed [3].

CMD type 1A is one of the most common forms of CMD and is typically characterized by elevated levels of creatine kinase. It is caused by a mutation of the LAMA2 gene, resulting in varied clinical symptoms depending on the onset of the disease, whether it is early or late. Notably, intellectual development remains unaffected in this condition.

In this case, the disease manifested at an early age, and the phenotype was characterized by hypotonia, predominantly proximal weakness, and contractures in the upper limbs at the elbow level, as well as in the lower limbs at the hips and knees. These manifestations resulted in functional motor limitations, affecting mobility and the use of the upper limbs. However, no alterations were observed in communication, swallowing, or cognitive function. The patient also presented comorbidities such as hip dysplasia, neuromuscular scoliosis, and obstructive sleep apnea-hypopnea syndrome.

What is particularly noteworthy in the case we are presenting is the severity of the contractures, which were the main cause of disability. Despite decreased muscle strength, the patient was mobile against gravity and demonstrated moderate endurance within the joint range limited by the contractures. Although activities related to gross motor function are impaired, significantly limiting technical transfers, the child maintains a good level of independence in many self-care activities such as feeding, dressing, and grooming.

Family history plays a crucial role in identifying the disease, which presents with weakness and proximal muscle contractures that limit walking. In addition to motor milestones delay, patients may exhibit hip pathology and respiratory complications. Therefore, it is imperative to conduct genetic studies, including sequencing of the LAMA2 gene. In this case, we discovered a previously unreported mutation variant, c. 1854_1861dup (p. Leu621Hisfs*7), found in homozygosity.

Weakness and contractures are slowly progressive, and most patients do not achieve independent ambulation. Facial weakness and jaw contractures disrupt normal feeding, resulting in failure to thrive. Most patients require nutrition support at an early age. Cardiac involvement is rare [8]. there are descriptions of impaired cardiac rhythm or left ventricular function, especially in adulthood, recommending follow-up with ultrasound and 24-hour Holter ECG regularly [9].

Cases of refractory epilepsy and mental retardation are much rarer and may show supra and infrastructural abnormalities on MRI, especially polymicrogyria or occipital cerebral agyria [10]. On the other hand, neurophysiology techniques (potential evoked, EMG) show signs of damage to central myelin and the peripheral disease that remains pauci or clinically asymptomatic [11].

Complementary to the diagnostic process, ultrasonography should also be considered due to its accessibility and rapid implementation at the patient's bedside. It can provide valuable information about the involvement of muscle tissue and potential disease progression during clinical follow-ups or during the initial evaluation of patients presenting with weakness, hypotonia, and contractures, which require a comprehensive diagnostic assessment for CMD.

Muscle imaging by ultrasonography and MRI are essential noninvasive tools to guide the differential diagnosis in patients with CMD and congenital myopathy. They can guide the rational selection of muscle for biopsy [8]. Although the majority of patients described with mutations LAMA2 do not present intellectual impairment; there is an anomaly very conspicuous in the supratentorial white matter, visible in the T2, Flair19, or STIR sequences, especially after six months [9]. 

In a study nationwide in China, one hundred and forty-nine patients were classified as LAMA2-related CMD (36.4% of the whole CMD cohort). All had a phenotype consistent with the classic course of the typical phenotype reported for LAMA2-related CMD, including white matter hyperintensity on brain MRI. Of those patients who consented to genetic testing (n = 125), mutations were found in 120 of the 125, to gen locus 6q22-23, and no mutation was found in 29 patients [12].

Deletion of exon 4 was detected in 10 alleles of eight patients, these patients are Han Chinese and were found to have the same haplotype and sequence at the breakpoint junctions, suggesting that exon 4 deletion is a founder mutation in Chinese Han and a mutation hotspot. In five patients, only one mutation was found, and MLPA and DNA array-based analyses are currently underway to detect copy number variation [13].

Given the physical, social, and adaptive participation commitment due to the disease, the patient receives physical and occupational rehabilitation management twice a week and psychological support once a week. It has shown improvement in musculoskeletal pain and control in the progression of muscle retractions. He is undergoing multidisciplinary follow-up by specialties such as orthopedics, neurology, endocrinology, pulmonology, child psychiatry, and physical medicine and rehabilitation.

## Conclusions

CMD resulting from merosin deficiency is characterized by the absence of laminin alpha-2. The clinical presentation and severity of the phenotype are associated with early onset of the disease. Patients with mutations in the LAMA2 gene exhibit either complete absence or partial reduction of laminin alpha-2 on staining. The precise prediction of the clinical phenotype based on the level of protein expression remains uncertain, and there have been reported cases of patients with laminin alpha-2 deficiency who develop seizures, with or without structural brain alterations. In this disease, there must be close follow-up and multidisciplinary specialized medical follow-up since it is a rare pathology that requires multiple interventions to improve quality of life and prognosis.

Ultrasonography can serve as a valuable tool to complement the clinical, immunohistochemical, and pathological findings, providing support for follow-up care or aiding in the diagnostic process for patients with CMD. Additionally, sequencing the LAMA2 gene revealed the presence of the c. 1854_1861dup (p. Leu621Hisfs*7) mutation in homozygosity. The clinical findings mentioned above are associated with the phenotype observed in this specific mutation.
